# Scattering from a finite-length closed perfect electric conducting circular cylinder: a regularized full-wave analysis

**DOI:** 10.1098/rsta.2024.0346

**Published:** 2025-08-14

**Authors:** Fulvio Schettino, Francesca Di Murro, Mario Lucido

**Affiliations:** ^1^ Department of Electrical and Information Engineering ‘Maurizio Scarano’, University of Cassino and Southern Lazio, Cassino, Lazio, Italy; ^2^ EUt+ Institute of Nanomaterials and Nanotechnologies-EUTINN, European University of Technology, Cassino, Italy

**Keywords:** electromagnetic scattering, analytical regularization, method of moments

## Abstract

In this paper, a regularized full-wave analysis of electromagnetic scattering from a finite-length closed perfect electric conducting circular cylinder is presented. By exploiting the azimuthal symmetry of the problem, the classical electric field integral equation is reduced to an infinite set of systems of one-dimensional integral equations in the spectral domain and solved by applying the Galerkin method with expansion functions reconstructing the physical behaviour of the unknown induced surface current density. In this way, regularization and quick convergence are achieved. Comparisons with the results in the literature and the ones obtained by means of commercial software CST Microwave Studio Suite are presented, showing the effectiveness of the proposed method.

This article is part of the theme issue ‘Analytically grounded full-wave methods for advances in computational electromagnetics’.

## 1. Introduction

The plane-wave scattering from an infinite-length perfect electric conducting (PEC) circular cylinder can be considered as a classical electromagnetic problem addressed in almost all the textbooks in this field. On the contrary, the analysis of a finite-length closed circular cylinder has been receiving little attention from the scientific community, which can be intuitively explained by its tremendously more complicated nature. As a matter of fact, the first example of investigation assumes that the finite-length circular cylinder is very thin to neglect the azimuthal variation of the fields [1]. In other papers, the authors suppose that the PEC circular cylinder is long enough with respect to the wavelength such that the surface current density on the finite-length cylinder can be approximated by that on an infinitely long one [2,3]. Most of the literature removing such limitations is substantially focused on the analysis of an open finite-length PEC circular cylinder [4–8]. To the best of the authors’ knowledge, the plane-wave scattering from a closed finite-length PEC circular cylinder has been addressed only by Tsuji & Shigesawa [9], applying the equivalent source method and supposing rounded wedges, by Stevens & Martens [10], using a method based on the magnetic field integral equation (IE), point-matching and a special choice of the expansion functions near the edges, and by Ross [11], focusing on the radar cross section obtained by means of physical optics, Wu’s series and empirical formulas. What is clear, when reading the quoted papers, is that the formulation of the boundary value problem for the Maxwell equations at hand in terms of IEs is widely preferred because of the advantages of incorporating the radiation condition of the field into the kernel asymptotic behaviour and of handling equations and unknowns defined in a finite domain [12]. Moreover, the uniqueness of the solution is guaranteed by the local power boundedness condition of the field at every frequency except those of possible internal resonances [13].

Unfortunately, the IEs thus obtained usually have singular kernels; hence, the existence of a solution cannot be generally stated [14]. In addition, even if the solution exists, no theorems can demonstrate the convergence of a general discretization scheme, and a finer and finer discretization can lead to ill-conditioned matrices to be inverted [14]. A strategy to overcome the aforementioned issues consists of transforming the equation at hand into a Fredholm second-kind IE by analytically inverting a suitable singular part of the integral operator [15,16]. Of course, after performing the analytical regularization of the integral operator, a suitable discretization maintaining the nature of the new equation and, subsequently, the truncation of the obtained infinite matrix equation are needed to achieve an approximate solution to the problem. An alternative regularizing approach adopted to deal with the above-mentioned problems consists of finding a set of orthonormal eigenfunctions of the most singular part of the integral operator to be used in a Galerkin discretization scheme. In this way, both regularization and discretization are combined in a single procedure, thus obtaining a Fredholm second-kind matrix equation without the need for the explicit Fredholm second-kind IE. More generally, such an approach properly works, i.e. Fredholm theory can be applied, even when the discretized counterpart of the singular part of the integral operator is simply invertible (with a continuous two-side inverse) and not necessarily diagonal, and the residual part is a compact operator on a suitable sequence space [17]. Such a procedure, called the method of analytical preconditioning (MAP) [18], has been widely used to solve propagation, radiation and scattering problems [19–23].

This paper deals with electromagnetic scattering from a finite-length closed PEC circular cylinder with flat end caps (hereafter also referred to as discs). The problem is initially formulated in terms of the electric field IE. Subsequently, the revolution symmetry of the scatterer is exploited by expanding the fields in a Fourier series. Hence, taking advantage of the orthogonality property of azimuthal harmonics, an infinite set of independent systems of one-dimensional IEs for the surface current densities (hereinafter, without risk of ambiguity, simply denoted as currents) induced on the discs and lateral surface is devised. Concerning that point, benefits arise from the choice of a suitable formulation in the spectral domain (the vector Fourier transform domain is considered for the contribution due to the lateral surface, whereas the vector Hankel transform (VHT) domain is preferred for the discs’ contributions). As a matter of fact, the discretization of the obtained systems of IEs is carried out according to MAP by selecting expansion functions with a closed-form spectral-domain counterpart, thus leading to a closed-form expression of the convolution integrals resulting from Galerkin projection, i.e. to a coefficient matrix of one-dimensional improper integrals efficiently numerically evaluable by means of an analytical procedure based on the extraction of the kernels’ asymptotic behaviour. Moreover, the choice of suitable expansion functions, specifically devised to reconstruct the physical behaviour of the surface current densities at each truncation order when right-angle wedge scatterers are involved, allows us to obtain good approximations of the exact solution of the problem by solving matrix equations of not large order. Numerical results and comparisons with the commercial software CST Microwave Studio are provided to show the effectiveness of the proposed method.

## 2. Formulation of the problem

**Figure 1.**
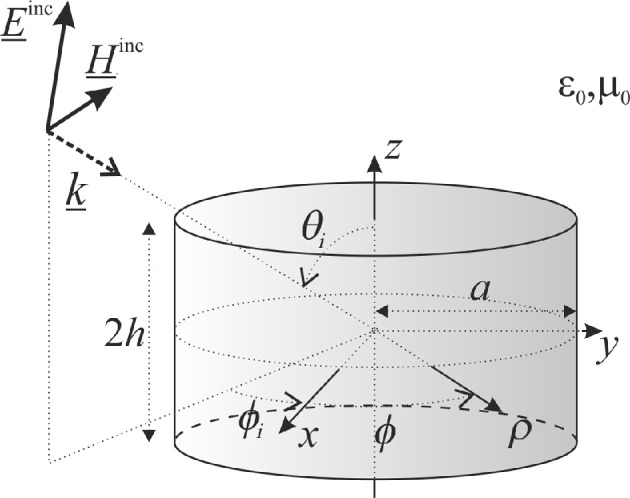
Geometry of the problem, incident plane wave and adopted notation.

Let us consider a closed finite-length PEC circular cylinder in free space, of radius a and overall length 2h, as sketched in figure 1: the cylinder is centred with respect to the coordinate system (x,y,z), its axis being along the z axis. The usual cylindrical coordinate system (ρ,ϕ,z) and spherical coordinate system (r,θ,ϕ) will also be employed in the following. A plane wave is impinging onto the cylinder with direction $\left(\theta_{i},\phi_{i}\right)$. The plane wave can be either transverse electric (TE) or transverse magnetic (TM) polarized with respect to the z axis. In the following, $\phi_{i}=0$ is assumed, without any loss of generality. Moreover, the analysis is developed in the frequency domain, assuming a time dependence $e^{j\omega t}$.

Due to revolution symmetry, the Fourier series expansion of all the involved quantities will be exploited, and the azimuthal index n will be understood and omitted for the sake of simplicity of notation. In addition, for each harmonic, the radial and azimuthal components of the field and the discs’ currents, in the following indicated as transverse components (with respect to the z axis), will be gathered in vector notation as


(2.1) $$F^{\left(n\right)}(\rho ,z)\equiv F(\rho ,z)=\left[\begin{array}{c} F_{\rho }(\rho ,z)\\ -jF_{\phi }(\rho ,z) \end{array}\right].$$

The closed cylinder at hand can be seen as a collection of two circular discs and a hollow finite-length circular cylinder: superscripts 1, 2 and L will then be used to indicate quantities related to the top disc, the bottom disc and the lateral surface, respectively. The (total) scattered field can then be written as the superposition of the fields generated by the currents induced on the three involved scatterers. In this section, the spectral-domain integral expressions of the scattered electric field contributions due to these scatterers are briefly summarized.

### (a) Circular disc in free space

The TE field due to the current induced on a PEC disc centred at the origin of the reference system by an incident field can be written as [24]


(2.2) $$E_{t}(\rho ,z)=-\frac{\zeta_{0}}{2}\int_{0}^{\infty }H_{n}(w\rho )G(w){\tilde{J}}_{d}(w)e^{-j|z|R(w)}wdw,$$

where $\zeta_{0}=\sqrt{\mu_{0}/\epsilon_{0}}$ and $k_{0}=\omega \sqrt{\mu_{0}\epsilon_{0}}$ are the free space impedance and wavenumber, respectively, ω is the angular frequency, $R(w)=\sqrt{k_{0}^{2}-w^{2}}=-j\sqrt{w^{2}-k_{0}^{2}}$, and the kernel G(w) is defined as


(2.3) $$G(w)=\left[\begin{array}{cc} K^{TM}(w) & 0\\ 0 & K^{TE}(w) \end{array}\right]=\left[\begin{array}{cc} \frac{R(w)}{k_{0}} & 0\\ 0 & \frac{k_{0}}{R(w)} \end{array}\right].$$

In (2.2), ${\tilde{J}}_{d}(w)$ is the VHT of the current density induced on the disc, $J_{d}(\rho )$, defined as


(2.4) $$\begin{array}{r} {\tilde{J}}_{d}(w)=\int_{0}^{\infty }H_{n}(w\rho )J_{d}(\rho )\rho d\rho , \end{array}$$

where the kernel of VHT is defined as


(2.5) $$H_{n}(w\rho )=\left[\begin{array}{cc} J_{n}^{\prime }(w\rho ) & \frac{n}{w\rho }J_{n}(w\rho )\\ \frac{n}{w\rho }J_{n}(w\rho ) & J_{n}^{\prime }(w\rho ) \end{array}\right],$$

$J_{n}(\cdot )$ being the Bessel function of the first kind and order n and the apex representing the derivative with respect to the argument. Some useful properties of the VHT can be found in [25]. The component along the z axis of the scattered electric field can be written as


(2.6) $$E_{z}(\rho ,z)=-j\frac{\zeta_{0}}{2}\mathrm{sgn}\;(z)\int_{0}^{\infty }J_{n}(w\rho )G_{z}^{T}(w){\tilde{J}}_{t}(w)e^{-j|z|R(w)}wdw,$$

where sgn(⋅) is the signum function, the superscript T represents the transpose and


(2.7) $$G_{z}(w)=\left[\begin{array}{c} w/k_{0}\\ 0 \end{array}\right].$$

In the above-given expressions, z has to be replaced by z−h and z+h when referring to the flat end caps of the cylinder.

### (b) Hollow finite-length circular cylinder in free space

The scattering by a hollow finite-length circular cylinder has been analysed in [8]. The expressions of the scattered electric field are reported here in a slightly different form in order to be consistent with the notation introduced in this paper for the transverse components. For the hollow cylinder, we can write


(2.8) $$\begin{array}{r} E_{t}(\rho ,z)=j\frac{\zeta_{0}}{4}\int_{-\infty }^{\infty }H_{n}(\rho R(u))K(u){\tilde{J}}^{\left(L\right)}(u)e^{-juz}du, \end{array}$$

where the Fourier transform of the current has been introduced, i.e.


(2.9) $${\tilde{J}}^{\left(L\right)}(u)=\left[\begin{array}{c} {\tilde{J}}_{\phi }^{\left(L\right)}(u)\\ {\tilde{J}}_{z}^{\left(L\right)}(u) \end{array}\right]=\frac{1}{2\pi }\int_{-\infty }^{\infty }\left[\begin{array}{c} J_{\phi }^{\left(L\right)}(z)\\ J_{z}^{\left(L\right)}(z) \end{array}\right]e^{juz}dz$$

and the kernel K(u) is defined as


(2.10) $$K(u)=\left[\begin{array}{cc} K_{\rho \phi }(u) & K_{\rho z}(u)\\ K_{\phi \phi }(u) & K_{\phi z}(u) \end{array}\right]=\left[\begin{array}{cc} \frac{nu^{2}}{ak_{0}}\frac{H_{n}^{\left(2\right)}(aR(u))}{R(u)} & \frac{u}{k_{0}}H_{n}^{\left(2\right)}(aR(u))R(u)\\ k_{0}H_{n}^{(2{)}^{\prime }}(aR(u)) & 0 \end{array}\right],$$

$H_{n}^{\left(2\right)}(\cdot )$ being the Hankel function of second kind and order n. The component along the z axis of the scattered electric field can be written as


(2.11) $$E_{z}(\rho ,z)=-\frac{\zeta_{0}}{4}\int_{-\infty }^{\infty }J_{n}(\rho R(u))K_{z}^{T}(u){\tilde{J}}^{\left(L\right)}(u)e^{-jzu}du,$$

where


(2.12) $$K_{z}(u)=\left[\begin{array}{c} \frac{nu}{ak_{0}^{2}}\\ 1-\frac{u^{2}}{k_{0}^{2}} \end{array}\right]k_{0}H_{n}^{\left(2\right)}(aR(u)).$$

### (c) System of integral equations

A system of IEs can now be obtained by imposing the boundary conditions on the surface of the close cylinder. Considering the Fourier series expansion of a plane wave [26], the following system is readily obtained


(2.13) $$\begin{array}{r} \left\{ \begin{array}{l} E_{t}^{\left(1\right)}(\rho ,\pm h)+E_{t}^{\left(2\right)}(\rho ,\pm h)+E_{t}^{\left(L\right)}(\rho ,\pm h)=-E_{t}^{inc}(r,\pm h)\;\rho \leq a\\ E_{\phi }^{\left(1\right)}(a,z)+E_{\phi }^{\left(2\right)}(a,z)+E_{\phi }^{\left(L\right)}(a,z)=-E_{\phi }^{inc}(a,z)\;\;|z|\leq h\\ E_{z}^{\left(1\right)}(a,z)+E_{z}^{\left(2\right)}(a,z)+E_{z}^{\left(L\right)}(a,z)=-E_{z}^{inc}(a,z)\;\;|z|\leq h. \end{array}\right. \end{array}$$

System (2.13) can be solved by resorting to suitable expansion functions in a Galerkin scheme. It is worth recalling that an instance of system (2.13) has to be solved for each index n. Hence, the boundary value problem at hand has been formulated in terms of an infinite set of independent systems of one-dimensional IEs.

## 3. Proposed solution

### (a) Physical behaviour of the currents

In order to solve the IEs in (2.13), the functions proposed in [27] and [8] to be used in a Galerkin scheme are no longer appropriate as the edge behaviour of the currents has changed with respect to the infinitesimally thin conductor. In the present case, the scatterer exhibits right-angle wedges, which require a different edge behaviour. In particular, Meixner’s conditions [28] allow us to state that the general harmonic of the current component parallel to the edge and the derivative of the current component perpendicular to the edge must exhibit an edge behaviour given by


(3.1) $$\begin{array}{r} J_{||},\frac{\partial J_{⊥}}{\partial \delta }\;\underset{\delta \overset{}{\to }0}{∼}\;\delta^{\frac{\psi -\pi }{2\pi -\psi }}, \end{array}$$

where δ is the distance from the edge. In the present case, since ψ=π/2, the leading behaviour of the parallel and the derivative of the perpendicular components of the current is given by $\delta^{-1/3}$. Moreover, the current component perpendicular to the edge does not vanish, but it must be continuous over the wedge, assuming a behaviour of the kind $c+d\;\delta^{2/3}$. The current components’ behaviour, together with the discs current components’ *n*-th harmonic behaviour around the discs’ centre, $\rho^{\left||n|-1\right|}$, properly defines the functional spaces to which the currents belong.

We want to solve the problem by resorting to the Helmholtz decomposition of $J-{\hat{J}}_{⊥}$, that is, the general induced current deprived of a suitable function, which will be defined later, taking into account the non-vanishing behaviour of the currents’ components perpendicular to the edges. To this purpose, two potentials, $\phi_{C}$ and $\phi_{D}$, related to the curl-free part, $J_{C}$, and to the divergence-free part, $J_{D}$, of $J-{\hat{J}}_{⊥}$, respectively, are introduced, i.e.


(3.2) $$\begin{array}{r} J-{\hat{J}}_{⊥}=J_{C}+J_{D}=\nabla_{s}\Phi_{C}+\hat{n}\times \nabla_{s}\Phi_{D}, \end{array}$$

where $\hat{n}$ is the unit normal to the surface where the current is located, either the discs or the lateral surface of the cylinder, $\nabla_{s}$ is the surface gradient.^1^ For our purpose, what is relevant is to mention that, since the current is related to such potentials by means of derivatives, it is easy to recognize that the edge behaviour of $\Phi_{\eta }$, where η stands for C or D, is of the kind $\delta^{p_{\eta }}$ with $p_{D}=2/3$ and $p_{C}=5/3$, respectively. We are ready to introduce the expansion functions that can be used to solve the system (2.13).

### (b) Basis functions of the potentials

The potential functions related to the *i*-th disc are expanded in the following series:


(3.3) $$\begin{array}{r} \Phi_{\eta }^{\left(i\right)}(\rho )=c_{-1}^{\left(i,\eta \right)}(1-\delta_{n,0})f_{-1}^{\;(\eta )}(\rho )+\sum_{q=0}^{\infty }c_{q}^{\left(i,\eta \right)}f_{q}^{\;(\eta )}(\rho ), \end{array}$$

where $\delta_{n,0}$ is the Kronecker symbol and $c_{q}^{\left(i,\eta \right)}$ with q≥−1 are unknown coefficients. In (3.3)


(3.4) $$f_{-1}^{\;(\eta )}(\rho )=\frac{(\text{sgn}\;n{)}^{n}\xi_{-1}^{\left(\eta \right)}}{|n|2^{p_{\eta }}a^{2}\Gamma \left(p_{\eta }\right)}\left\{ \begin{array}{cc} {\left(\frac{\rho }{a}\right)}^{|n|}{\;}_{2}F_{1}\left(1-p_{\eta },|n|;|n|+1;\frac{\rho^{2}}{a^{2}}\right) & \rho \leq a\\ \frac{\Gamma \left(p_{\eta }\right)\Gamma (|n|+1)}{\Gamma \left(|n|+p_{\eta }\right)}{\left(\frac{\rho }{a}\right)}^{-|n|} & \rho >a \end{array}\right.,$$

whereas for q≥0,


(3.5) $$f_{q}^{\;(\eta )}(\rho )=\left\{ \begin{array}{cc} \frac{\xi_{q}^{\left(\eta \right)}\Gamma \left(q+1\right)(\text{sgn}\;\left(n\right){)}^{n}}{2^{p_{\eta }}a^{2}\Gamma \left(q+p_{\eta }+1\right)}{\left(\frac{\rho }{a}\right)}^{|n|}{\left(1-\frac{\rho^{2}}{a^{2}}\right)}^{p_{\eta }}P_{q}^{\left(|n|,p_{\eta }\right)}\left(1-2\frac{\rho^{2}}{a^{2}}\right) & \rho \leq a\\ 0 & r>a \end{array}\right.,$$

where $\xi_{q}^{\left(\eta \right)}=\sqrt{2(2q+n+p_{\eta }+1)}$ is a suitable normalization quantity. In (3.4), Γ(⋅), ${,}_{2}F_{1}(\cdot ,\cdot ;\cdot ;\cdot )$ and $P_{q}^{\left(\cdot ,\cdot \right)}(\cdot )$ denote the Gamma and the hypergeometric functions and the Jacobi polynomial of order q, respectively. By setting $\xi_{-1}^{\left(D\right)}c_{-1}^{\left(i,D\right)}=j\xi_{-1}^{\left(C\right)}c_{-1}^{\left(i,C\right)}/2(n+p_{D})$, when substituting functions defined by (3.4) into (3.2), we get


(3.6) $$\begin{array}{r} \frac{d}{d\rho }f_{-1}^{\;(C)}(\rho )-\frac{jn}{\rho }\frac{j\xi_{-1}^{\left(C\right)}}{2(n+p_{D})\xi_{-1}^{\left(D\right)}}f_{-1}^{\left(D\right)}(\rho )=\left\{ \begin{array}{cc} \frac{(|n|+4/3)\xi_{-1}^{\left(C\right)}}{(|n|+3/2)2^{5/3}\Gamma (5/3)a^{3}}{\left(\frac{\rho }{a}\right)}^{|n|-1}{\left(1-\frac{\rho^{2}}{a^{2}}\right)}^{2/3} & \rho <a\\ 0 & \rho >a \end{array}\right. \end{array}$$

(3.7) $$\begin{array}{r} \frac{jn}{\rho }f_{-1}^{\;(C)}(\rho )+\frac{j\xi_{-1}^{\left(C\right)}}{2(n+p_{D})\xi_{-1}^{\left(D\right)}}\frac{d}{d\rho }f_{-1}^{\;(D)}(\rho )=\left\{ \begin{array}{cc} \frac{(|n|+4/3-|n|\rho^{2}/a^{2})\xi_{-1}^{\left(C\right)}}{(|n|+2/3)2^{5/3}\Gamma (5/3)a^{3}}{\left(\frac{\rho }{a}\right)}^{|n|-1}{\left(1-\frac{\rho^{2}}{a^{2}}\right)}^{-1/3} & \rho <a\\ 0 & \rho >a \end{array}\right., \end{array}$$

which reconstruct the physical behaviour around the origin and at the edge of the current components along ρ, deprived by the non-vanishing value on the wedge, and along ϕ, respectively. It is not difficult to verify that the VHTs of the curl-free and divergence-free components of the discs’ currents can be expressed in closed form, and are given by


(3.8) $$VHT[J_{C}^{\left(i\right)}]=\sum_{q=-1+\delta_{n,0}}^{\infty }c_{q}^{\left(i,C\right)}\left[\begin{array}{c} w{\tilde{f}}_{q}^{\;(C)}(w)\\ 0 \end{array}\right]$$

and


(3.9) $$VHT[J_{D}^{\left(i\right)}]=\sum_{q=-1+\delta_{n,0}}^{\infty }c_{q}^{\left(i,D\right)}\left[\begin{array}{c} 0\\ w{\tilde{f}}_{q}^{\;(D)}(w) \end{array}\right],$$

where


(3.10) $${\tilde{f}}_{q}^{\left(\eta \right)}(w)=\int_{0}^{\infty }f_{q}^{\left(\eta \right)}(\rho )J_{n}(w\rho )\rho d\rho =\xi_{q}^{\left(\eta \right)}\frac{J_{|n|+2q+p_{\eta }+1}(wa)}{(wa{)}^{\left(p_{\eta }+1\right)}}.$$

Similarly, with reference to the lateral surface, we can use


(3.11) $$\begin{array}{r} \Phi_{\eta }^{\left(L\right)}(z)=d_{-1}^{\left(L,\eta \right)}(1-\delta_{n,0})g_{-1}^{\left(L,\eta \right)}(z)+\sum_{q=0}^{\infty }d_{q}^{\left(L,\eta \right)}g_{q}^{\left(L,\eta \right)}(z), \end{array}$$

where $d_{q}^{\left(L,\eta \right)}$ with q≥−1 are unknown coefficients, and the functions appearing in the first term on the right-hand side are defined as


(3.12)g−1(L,C)(z)=−d−1(L,C)∫−∞∞J1/6(hu)(au)1/6ne−juzn2+u2a2du+d−1(L,D)∫−∞∞J7/6(hu)(au)1/6e−juzn2+u2a2du,(3.13)g−1(L,D)(z)=d−1(L,D)∫−∞∞J7/6(hu)(au)7/6ne−juzn2+u2a2du+d−1(L,C)∫−∞∞J1/6(hu)(au)1/6uae−juzn2+u2a2du.

An explicit expression of the above integrals can be obtained for |z|>h by applying formulas in [29, eqns 6.718.3-4], showing an exponential decay with respect to z, whereas when substituting (3.12) and (3.13) into (3.2), we obtain


(3.14)ddzg−1(L,C)(z)−jnag−1(L,D)(z)=−jad−1(L,D)∫−∞∞J7/6(uh)(au)7/6e−juzdu==−jahd−1(L,D){(ha)7/6π21/6Γ(5/3)(1−z2h2)2/3|z|<h0|z|>h(3.15)jnag−1(L,C)(z)+ddzg−1(L,D)(z)=−jad−1(L,C)∫−∞∞J1/6(uh)(au)1/6e−juzdu==−jahd−1(L,C){(ha)1/625/6πΓ(2/3)(1−z2h2)−1/3|z|<h0|z|>h,

which reconstructs the edge behaviour of the current components along ϕ and along z, deprived by the non-vanishing value on the wedge, respectively. The closed-form expressions of the integrals appearing in (3.14) and (3.15) have been obtained by particularizing the more general formula [29]


(3.16) $$\int_{-\infty }^{\infty }\frac{J_{m+\nu }(hu)}{(hu{)}^{\nu }}e^{-juz}du=\left\{ \begin{array}{cc} \frac{2^{\nu }\Gamma (m+1)\Gamma (\nu )}{j^{m}h\Gamma (m+2\nu )}{\left(1-\frac{z^{2}}{h^{2}}\right)}^{\nu -1/2}C_{m}^{\left(\nu \right)}\left(\frac{z}{h}\right) & |z|<h\\ 0 & |z|>h \end{array}\right..$$

The remaining expansion functions in (3.11) are defined as follows:


(3.17) $$g_{q}^{\left(L,\eta \right)}(z)=\{\begin{array}{cc} \frac{\chi_{q}^{\left(\eta \right)}2^{\left(p_{\eta }+1/2\right)}\Gamma (q+1)\Gamma (p_{\eta }+1/2)}{j^{q}h\Gamma (q+2p_{\eta }+1)}{\left(1-\frac{z^{2}}{h^{2}}\right)}^{p_{\eta }}C_{q}^{\left(p_{\eta }+1/2\right)}\left(\frac{z}{h}\right) & |z|<h\\ 0 & |z|>h \end{array}\;,$$

where $\chi_{q}^{\left(\eta \right)}=\sqrt{q+p_{\eta }+1/2}$ is a suitable normalization quantity.

Closed-form expressions for the Fourier transforms ${\tilde{g}}_{q}^{\left(L,\eta \right)}(u)$ of the expansion functions in (3.11) can be immediately obtained starting from (3.12), (3.13) and (3.16) as (q≥−1)


(3.18) $$\begin{array}{lrr} & {\tilde{g}}_{q}^{\left(\eta \right)}(w)=\frac{1}{2\pi }\int_{-\infty }^{\infty }g_{q}^{\left(L,\eta \right)}(z)e^{juz}dz=\chi_{q}^{\left(\eta \right)}\frac{J_{q+p_{\eta }+1/2}(hu)}{(hu{)}^{\left(p_{\eta }+1/2\right)}}. & \end{array}$$

### (c) Continuity condition

We can now discuss the functions supplementing the above-given expansion in order to guarantee the continuity across the edges, that is, the term $\underline{\hat{J}}{\;}_{⊥}$ in equation (3.2). With reference to the *i*-th disc, the following function can be added to the radial component of the current


(3.19) $${\hat{J}}_{\rho }^{\left(i\right)}(\rho )=\alpha_{i}\;{\hat{f}}_{\rho }(\rho )=\alpha_{i}\frac{1}{a^{3}}{\left(\frac{\rho }{a}\right)}^{|n|+1}\;\rho \leq a,$$

whereas with reference to the lateral cylinder, a simple linear function can be added to the longitudinal component of the current


(3.20) $${\hat{J}}_{z}^{\left(L\right)}(z)=\beta {\hat{g}}_{z1}(z)+\gamma {\hat{g}}_{z2}(z)=\frac{1}{h^{2}}\left(\beta \frac{z}{h}+\gamma \right)\;|z|\leq h,$$

where $\alpha_{i}$, β and γ have to be chosen so as to guarantee continuity over the wedges. To this purpose, it is worth remembering that, according to Meixner’s conditions, the ϕ component of the magnetic field is continuous over the wedges. This condition is reflected in the following equations, which relate the components of the currents perpendicular to the edges to each other


(3.21) $$J_{\rho }^{\left(1,2\right)}(\rho =a)=\mp J_{z}^{\left(L\right)}(z=\pm h)/(2\pi a).$$

As a result, the following relations between the coefficients in (3.18) and (3.19) can be readily established


(3.22)−α1=a22πh2β+γ2a(3.23)α2=a22πh2−β+γ2a.

To conclude, it is worth observing that even the VHT of the function in (3.19) and the Fourier transform of the ones in (3.20) can be simply expressed in the following closed-forms:


(3.24) $$\text{VHT}\left[\begin{array}{c} {\hat{f}}_{\rho }(\rho )\\ 0 \end{array}\right]=\left[\begin{array}{c} {\tilde{f}}_{\rho 1}(w)\\ {\tilde{f}}_{\rho 2}(w) \end{array}\right]=\left[\begin{array}{c} \frac{(\text{sgn}\;\left(n\right){)}^{n}}{a}\frac{|n|J_{|n|+1}(wa)-waJ_{|n|+2}(wa)}{(wa{)}^{2}}\\ \frac{(\text{sgn}\;\left(n\right){)}^{n+1}}{a}\frac{|n|J_{|n|+1}(wa)}{(wa{)}^{2}} \end{array}\right]$$

(3.25) $$\begin{array}{r} \frac{1}{2\pi }\int_{-h}^{h}\left[\beta {\hat{g}}_{z1}(z)+\gamma {\hat{g}}_{z2}(z)\right]e^{juz}dz=j\frac{h}{4a^{2}}\frac{1}{uh}\left[\alpha_{1}\left(e^{juh}-\frac{\sin uh}{uh}\right)+\alpha_{2}\left(e^{-juh}-\frac{\sin uh}{uh}\right)\right]. \end{array}$$

### (d) Matrix equation

Summarizing, we have introduced expansion functions with closed-form vector Hankel/Fourier transforms of all the components of the currents induced on the cylinder to be used in the Galerkin scheme. As a result, the spectral-domain formulation allows us to express the convolution integrals resulting from Galerkin projection in closed form. In this way, the elements of the obtained scattering matrix are one-dimensional integrals to be numerically computed. Except for the integrals of mutual contribution among the discs, whose integrands exhibit an exponential asymptotic decay related to their distance, the others are improper integrals of oscillating and asymptotically slowly decaying functions. A classical technique for the efficient evaluation of such kinds of integrals consists in the extraction of the asymptotic behaviour of the kernel [30]. In this way, the integrals of the extracted contributions can be expressed in closed form or evaluated once and for all independently of the frequency, whereas the residual integrals converge quickly. Therefore, the matrix equation can be written as follows:


(3.26) $$\begin{array}{lrr} & M\;x=M^{\prime }x+M^{\prime \prime }x=c, & \end{array}$$

where $M^{\prime }$ and $M^{\prime \prime }$ are the matrix operators of the extracted contributions and of the residual ones, respectively, and c is the free term vector. The scattering matrix M is symmetric due to reciprocity, and an explicit expression of all the elements, including some simplifying relationships among them, is provided in the electronic supplementary material. What is really interesting to note is that, by means of a strategy requiring simple but long algebraic manipulations (omitted here for the sake of brevity) that follows the reasoning proposed in [19], it is possible to show that $M^{\prime }$ is a positive definite and, hence, an invertible matrix operator, whereas $M^{\prime \prime }$ is a compact operator in the space of the square-summable sequences $l^{2}$. Since c belongs to the same space, Fredholm theory can be applied [17], which translates to the guaranteed convergence of the adopted discretization scheme to the exact solution of the problem. In other words, the accuracy of the approximate solution obtained by truncating the matrix equation can be increased by considering a larger number of expansion functions and/or azimuthal harmonics and is limited only by the machine precision.

## 4. Numerical results

In the previous section, it has been demonstrated that the discretization scheme adopted to solve the systems of IEs in (2.13) converges to the exact solution of the problem. In this section, we want to show that, besides this remarkable result, the proposed method is also effective. As a matter of fact, since the physical behaviour of the currents is properly reconstructed, accurate solutions can be achieved by solving small-sized truncated matrix equations. Moreover, the method is low time-consuming due to the speeding up of the numerical evaluation of the integrals of the coefficient matrix. The fast convergence of the proposed method in terms of both computation time and memory occupation will be proven by means of comparisons with the literature and CST-MWS and by introducing the following normalized truncation error:


(4.1) $$\begin{array}{r} err_{N}(Q)=\sqrt{\sum_{n=-N+1}^{N-1}{\left\|x_{Q+1}^{\left(n\right)}-x_{Q}^{\left(n\right)}\right\|}^{2}/\sum_{n=-N+1}^{N-1}{\left\|x_{Q}^{\left(n\right)}\right\|}^{2}}, \end{array}$$

where ||⋅|| is the usual Euclidean norm and $x_{Q}^{\left(n\right)}$ is the vector of the expansion coefficients of the *n*-th harmonic current components evaluated by using Q expansion functions. It must be pointed out that 2N−1, i.e. the number of azimuthal harmonics to be used, can be estimated following the same line of reasoning reported in [31]. It is worth underlying that the matrix equation (3.26), whose elements are described in the electronic supplementary material, can be readily implemented by the reader with the aid of a general quadrature routine, a routine for the evaluation of Bessel functions and a standard matrix equation solver. The numerical results proposed in this paper are obtained by means of an in-house software code, implemented in a MATLAB environment and based on an adaptive Gaussian quadrature routine, running on a laptop equipped with an Intel Core 7 150U 1.8 GHz processor and 32 GB of RAM. In all cases, an incident electric field $|E_{0}|=1\;\text{V/m}$ is assumed, unless otherwise specified.

**Figure 2.**
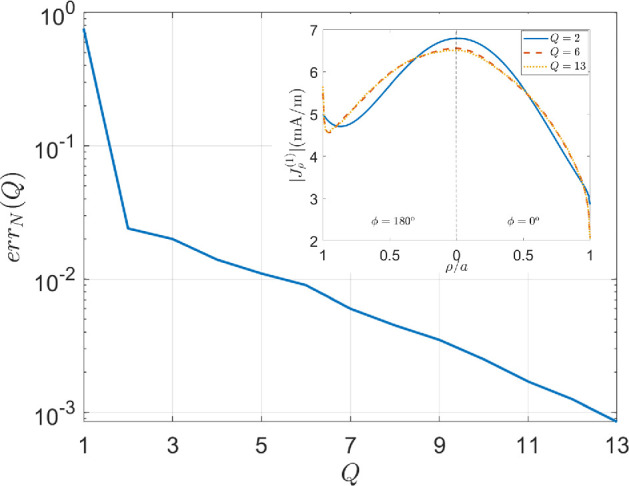
Error as a function of truncation order Q. The cylinder has dimensions a=h=λ/4, and the plane wave is impinging with $\theta_{i}=15^{\circ }$, $\phi_{i}=0^{\circ }$, $|E_{0}|=1\;\text{V/m}$ and TM polarization. In the inset, the amplitude of the radial component of the current on the top disc is plotted for different truncation orders Q.

As a case test, in figure 2, the normalized truncation error for a=h=λ/4 and an impinging plane wave with $\theta_{i}=15^{\circ }$, $\phi_{i}=0^{\circ }$, and TM polarization is shown for varying values of Q, assuming N=2 according to [31]. As can be observed, the error exhibits an asymptotic exponential decay. Moreover, it is less than $10^{-1}$, $10^{-2}$ and $10^{-3}$ for Q=2, 6 and 13, respectively. In the inset of the figure, the amplitude of the radial component of the current on the top disc, $\left|J_{\rho }^{\left(1\right)}\right|$, is plotted for Q=2, 6 and 13. It is interesting to observe that the currents reconstructed for Q=6 and Q=13 are almost indistinguishable, clearly showing the fast convergence of the proposed method. Such behaviour can be proven for all the current components of the considered case, as well as for cylinders of different sizes and different plane wave incidence angles and polarization. For this reason, henceforth, $err_{N}(Q)<10^{-2}$ is assumed as a convergence criterion.

**Figure 3.**
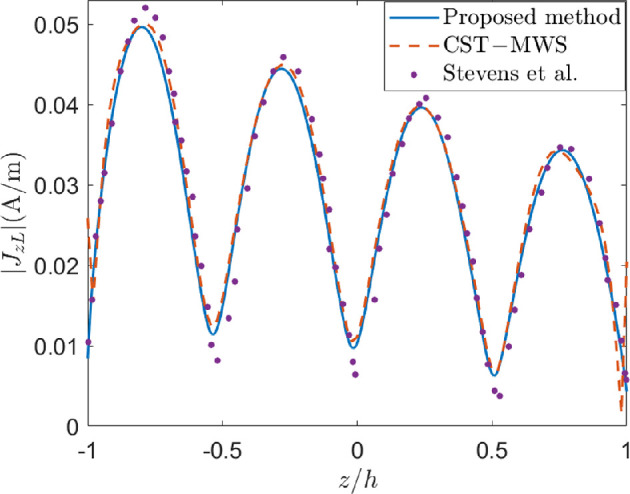
Longitudinal current on a cylinder with a=λ/74.2 and h=λ, and an impinging plane wave with incident angle $\theta_{i}=\pi /6$, $|H_{0}|=1\;\text{A/m}$ and TM polarization. Comparison with CST-MWS and results from [10]. N=2 and Q=8 have been used in the proposed method.

In figure 3, the amplitude of the longitudinal component of the current on the lateral surface of the cylinder, $\left|J_{z}\right|$, for $\phi =0^{\circ }$, a=λ/74.2 and h=λ, induced by an impinging TM-polarized plane wave with $\theta_{i}=30^{\circ }$, $\phi_{i}=0^{\circ }$ and amplitude of the incident magnetic field $|H_{0}|=1\;\text{A/m}$, has been reconstructed. Such behaviour agrees quite well with the results provided by Stevens & Martens [10] and obtained by means of the point-matching method applied to a magnetic field IE and the introduction of suitable functions retaining the current behaviour on the wedges. Moreover, the comparison with the current provided by CST-MWS shows a very good agreement. It is worth observing that a normalized truncation error less than $10^{-2}$ is achieved for N=2 and Q=8 with a computation time of slightly more than 3 min, whereas CST-MWS requires 0.4 M mesh-cells and approximately 9 min to reconstruct the solution. As a result, the proposed method drastically outperforms CST-MWS in terms of computational resources.

**Figure 4.**
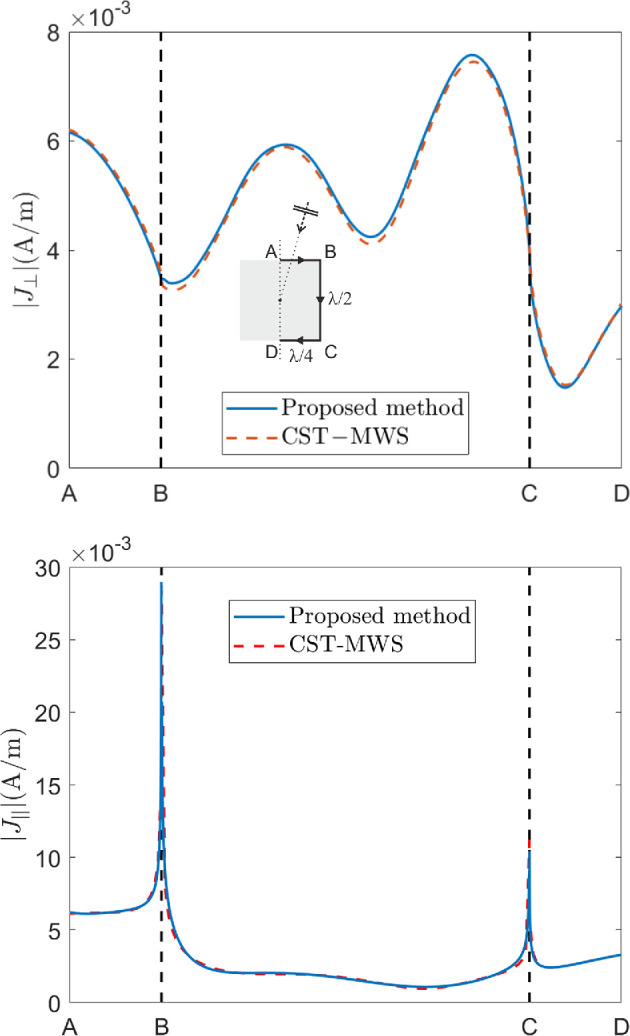
Current along the curve on the cylinder surfaces in the half-plane $\phi =0^{\circ }$ sketched in the inset, with a=λ/4 and h=λ/2, for TM incidence (above) and TE incidence (below) with $\theta_{i}=15^{\circ }$ and $|E_{0}|=1\;\text{V/m}$. N=2 and Q=8 have been used in the proposed method.

**Figure 5.**
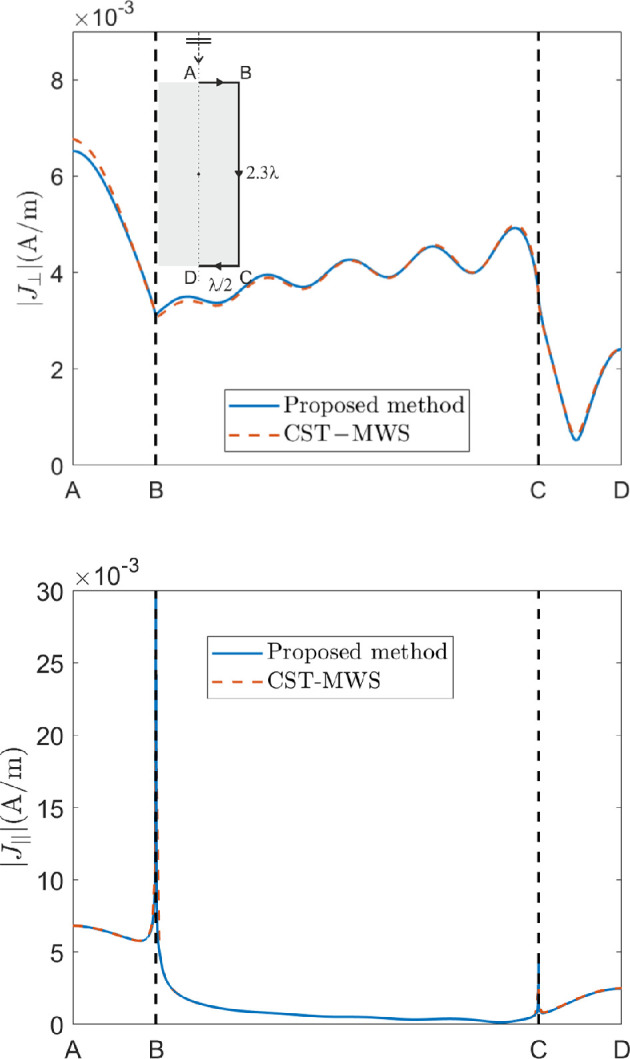
Current along the curve on the cylinder surfaces in the half-plane $\phi =0^{\circ }$ sketched in the inset, with a=λ/2 and h=1.15λ, for TM incidence (above) and TE incidence (below) with $\theta_{i}=0^{\circ }$ and $|E_{0}|=1\;\text{V/m}$. N=2 and Q=15 have been used in the proposed method.

Many different cases have been simulated, and the current behaviour for some of them is shown in the following and compared with the results obtained by CST-MWS. In figures 4 and 5, the amplitude of the current component perpendicular to the wedges, $\left|J_{⟂}\right|$, is plotted in the TM case, whereas the amplitude of the current component parallel to the wedge, $\left|J_{\left|\right|}\right|$, is plotted in the TE case. They are plotted over the curves on the cylinder surfaces in the half-plane $\phi =0^{\circ }$ sketched in the insets of figures. In figure 4 a cylinder with a=λ/4 and h=λ/2 and an incident plane wave with $\theta_{i}=15^{\circ }$, $\phi_{i}=0^{\circ }$ are considered. In this case, N=2 and Q=8 have been chosen with a computation time of less than 2 min, and the comparison with the results provided by CST-MWS, by using 3.4 M mesh-cells with a computation time of more than 16 min, shows a very good agreement. In figure 5, the current on a larger cylinder with a=λ/2 and h=1.15λ, and a plane wave normally impinging onto it ($\theta_{i}=0^{\circ }$), is reconstructed and compared with very good agreement with the results provided by CST-MWS. In this case, N=2 and Q=15 have been chosen with a computation time of approximately 3.5 min, whereas CST-MWS required 7.6 M mesh-cells with a computation time of 23.5 min. As expected, a higher, however reasonable, number of expansion functions is needed to reconstruct the solution due to the longer length of the cylinder with respect to the previously considered cases. On the contrary, a significantly higher memory occupation is needed to achieve a quite accurate solution by means of CST-MWS in this last case.

## 5. Conclusion

The plane-wave scattering from a finite-length closed PEC circular cylinder, formulated in terms of an infinite set of systems of singular IEs in the spectral domain for the azimuthal harmonics of the current on the cylinder surface, has been solved by means of MAP, thus obtaining a matrix equation at which Fredholm’s theory can be applied. The suitable choice of the expansion functions, reconstructing the physical behaviour of the field with a closed-form spectral-domain counterpart, has allowed to achieve highly accurate results with few expansion functions and azimuthal harmonics and a matrix equation of one-dimensional integrals quickly evaluable or made so by the application of the analytical asymptotic acceleration technique. The proposed numerical results and the comparisons with the literature and the commercial software CST-MWS have clearly shown the effectiveness of the proposed method in terms of both computation time and memory occupation.

## Data Availability

The proposed method, and specifically the matrix equation, can be readily implemented by the reader with the aid of a general quadrature routine, a routine for the evaluation of Bessel functions, and a standard matrix equation solver. The expressions of all the required elements of the matrix are explicitly reported in the electronic supplementary material [32].

## Footnotes

^1^
In surface gradient, the derivative with respect to ϕ is formally replaced by multiplication by jn due to the Fourier series expansion in cylindrical harmonics.
